# Proteomic Analysis of Antigen 60 Complex of *M. bovis* Bacillus Calmette-Guérin Reveals Presence of Extracellular Vesicle Proteins and Predicted Functional Interactions

**DOI:** 10.3390/vaccines7030080

**Published:** 2019-08-02

**Authors:** Khayriyyah Mohd Hanafiah, Norsyahida Arifin, Paul R. Sanders, Nurulhasanah Othman, Mary L. Garcia, David A. Anderson

**Affiliations:** 1 School of Biological Sciences, Universiti Sains Malaysia, Penang 11800, Malaysia; 2 Life Sciences, Macfarlane Burnet Institute, Melbourne, VIC 3004, Australia; 3 Institute for Research in Molecular Medicine, Universiti Sains Malaysia, Penang 11800, Malaysia

**Keywords:** mass spectrometry, tuberculosis, BCG, protein–protein interaction, biomarkers, extracellular vesicles

## Abstract

Tuberculosis (TB) is ranked among the top 10 causes of death worldwide. New biomarker-based serodiagnostics and vaccines are unmet needs stalling disease control. Antigen 60 (A60) is a thermostable mycobacterial complex typically purified from Bacillus Calmette-Guérin (BCG) vaccine. A60 was historically evaluated for TB serodiagnostic and vaccine potential with variable findings. Despite containing immunogenic proteins, A60 has yet to be proteomically characterized. Here, commercial A60 was (1) trypsin-digested in-solution, analyzed by LC-MS/MS, searched against *M. tuberculosis* H37Rv and *M.bovis* BCG Uniprot databases; (2) analyzed using STRING to predict protein–protein interactions; and (3) probed with anti-TB monoclonal antibodies and patient immunoglobulin G (IgG) on Western blot to evaluate antigenicity. We detected 778 proteins in two A60 samples (440 proteins shared), including DnaK, LprG, LpqH, and GroEL1/2, reportedly present in mycobacterial extracellular vesicles (EV). Of these, 107 were also reported in EVs of *M. tuberculosis*, and 27 key proteins had significant protein–protein interaction, with clustering for chaperonins, ribosomal proteins, and proteins for ligand transport (LpqH and LprG). On Western blot, 7/8 TB and 1/8 non-TB sera samples had reactivity against 37–50 kDa proteins, while LpqH, GroEL2, and PstS1 were strongly detected. In conclusion, A60 comprises numerous proteins, including EV proteins, with predicted biological interactions, which may have implications on biomarker and vaccine development.

## 1. Introduction

Tuberculosis (TB) persists as a top global cause of death, particularly in low-income areas [1]. *Mycobacterium tuberculosis* complex (MTB) are the causative agents of tuberculosis, and consist of several related pathogenic mycobacteria including *M. africanum*, *M. caprae* and *M.bovis* [2]. The latter typically infects cattle and its attenuated strain, Bacillus Calmette-Guérin (BCG), has been used as a live TB vaccine since 1921 [3]. However, the BCG affords poor protection for adults and is incompatible with immunocompromised individuals [4]. Furthermore, the diversity of pathogenic strains that cause TB and heterogeneous clinical manifestations across different subpopulations [5,6,7], reliance on laboratory-confined sputum-based diagnostics [8], and cumbersome treatment regimens [9], have complicated efforts to significantly decrease global TB transmission, particularly among the majority of TB-infected individuals living in resource-limited areas. Therefore, high priority research targets include discovery and development of antigen preparations for accurate biomarker-based non-sputum assays and improved vaccines that can be used to protect adults and immunocompromised/human-immunodeficiency virus (HIV)-infected individuals [8,10,11,12].

One particular candidate is antigen 60 (A60), a high molecular weight (HMW) thermostable macromolecular antigen (TMA) complex previously described as being present in mycobacterial cytoplasm [13,14], cell wall and extracellular matrix [15,16]. Typically extracted from BCG [17], A60 has been previously used in serological assays to diagnose TB and evaluated for vaccine potential [18,19,20]. Despite containing several immunogenic antigens [21], A60-based assays suffered severe limitations in diagnostic specificity, particularly in areas with high TB and HIV prevalence [7,18], resulting in their fall from favor. Early vaccine evaluations of A60 as a subunit TB vaccine candidate further demonstrated inferior protection compared to BCG vaccination [20].

Earlier studies on composition of the 10^3^–10^4^ kDa A60 employing immunological methods such as immunodiffusion and Western blot, as well as gas chromatography/mass spectrometry (GC/MS) has described the A60 as a lipoprotein-polysaccharide complex [22,23]. The complex reportedly comprises 30 antigens between 30–65 kDa in size including immunodominant heat-shock proteins such as GroEL2 and HspX [15,16,21], which are also present in old tuberculin and purified protein derivative (PPD), crude antigens used in the Mantoux skin test for diagnosing latent TB [24]. The presence of several immunogenic antigens in this complex [25] is presumed to be an artefact of cell lysis, resulting in proteins aggregating into micelles. However, recent reports of lipoglycan-and lipoprotein-containing membrane-bound bacterial extracellular vesicles (EV) produced by MTB and involved in host pathogen interactions [26] such as by directly regulating T cell activity through exosomes released by macrophages [27], raises the question of whether the HMW A60 may be associated with EVs.

The study of EVs in mycobacteria was historically neglected due to their lack of outer membrane and distinctive thick cell wall, precluding the possibility that membrane-derived vesicles would be released from such walls. However, production of EVs ranging from 50–300 nm in size are now accepted to be a conserved phenomenon across the Mycobacterium genus, observed in both medically important species such as MTB and BCG, as well as non-pathogenic environmental mycobacteria [28,29]. Proteomically, MTB and BCG EVs are known to be enriched in lipoproteins such as LpqH, LppX, LprA and PstS1 [29], a group of virulence-associated proteins able to interfere with antigen presentation, which increasingly appear to serve as MTB emissaries sent to modulate T cells of infected hosts towards less protective responses [27,30,31,32,33].

A key gap in characterization of A60, and addressing whether it is an artefact of cell lysis or instead has biological associations with EVs, is the fact that previous investigations relied on gel-based immunological methods, which were limited by available monoclonal antibodies. As a first step towards better understanding the origins and potential application of this antigenic preparation on future biomarker and vaccine discovery, this article highlights findings from a shotgun proteomic analysis of commercial BCG-derived A60, corresponding predictive protein–protein interactions between member antigens and evaluation of antigenicity using patient serum, which together indicate presence of functional protein–protein interactions within the A60 complex, including several proteins that have been described in mycobacterial EVs.

## 2. Materials and Methods

### 2.1. Trypsin Digestion

100 μg protein from two samples of commercially acquired A60 batch AT071002 (PBC Maes, Strasbourg, France) (A60 S1, A60 S2), which has been described as the peak exclusion fraction of lysate from log-phase grown BCG run through a Sepharose 6B size exclusion chromatography column (SEC) [34], were subjected to in-solution digestion using trypsin on separate days. Briefly, proteins were precipitated in ice-cold acetone overnight, then reduced using 10 mM tris(2-carboxyethyl) phosphine (Sigma Aldrich, St. Louis, MO, USA) (45 min, dark at 37 °C) and alkylated using 55 mM iodoacetamide (Sigma-Aldrich) (30 min, dark at room temperature (RT)). Samples were trypsin (Thermo Fisher Scientific, Waltham, MA, USA) digested at 1:50 enzyme: protein ratio (overnight, 37 °C), acidified to pH < 2 with formic acid, and desalted using Pierce C18 Spin Columns (Thermo Fisher Scientific) with flow-through passed back over the column twice. Proteins were eluted with 80% acetonitrile containing 0.1% trifluoroacetic acid, concentrated to 20 μL by SpeedVac^TM^ (Thermo Fisher Scientific) and stored at −80 °C prior to mass spectrometry analysis.

### 2.2. Mass Spectrometry (MS)

LC-MS/MS was performed using Orbitrap Lumos mass spectrometer (Thermo Fisher Scientific) fitted with nanoflow reversed-phase HPLC (Ultimate 3000 RSLC, Dionex). The nano-LC system was equipped with an Acclaim Pepmap nano-trap column and an Acclaim Pepmap RSLC analytical column. 1 μL of the peptide mix was loaded onto the enrichment (trap) column at an isocratic flow of 5 μL/min of 3% acetonitrile containing 0.1% formic acid for 6 min before the enrichment column was switched in-line with the analytical column. The eluents used for the LC were 0.1% *v*/*v* formic acid (solvent A) and 100% acetonitrile/0.1% formic acid *v*/*v*. The gradient used was 3% B to 20% B for 95 min, 20% B to 40% B in 10 min, 40% B to 80% B in 5 min and maintained at 80% B for the final 5 min before equilibration for 10 min at 3% B prior to the next sample. The mass spectrometer was equipped with a NanoEsi nano-electrospray ion source (Thermo Fisher Scientific) for automated MS/MS. High mass accuracy MS data were obtained in a data-dependent acquisition mode with the Orbitrap resolution set at 75,000 and the top-ten multiply charged species selected for fragmentation by higher-energy collisional dissociation (HCD) (single-charged and double-charged species were ignored). The ion threshold was set to 15,000 counts for MS/MS. The capillary electrophoresis (CE) voltage was set to 27. The resolution was set to 120,000 at MS1 with lock mass of 445.12003 with HCD Fragmentation and MS2 scan in ion trap. Top 3 s method was used to select species for fragmentation. Singly charged species were ignored and an ion threshold triggering at 1 × 10^4^ was employed. CE voltage was set to 1.9 kV.

### 2.3. Seroimmunological Analysis

#### 2.3.1. Serum Sample Population

Archived serum samples acquired from repositories managed by the Foundation for Innovative New Diagnostics (FIND) (25) were from Vietnamese HIV-negative eligible consenting (>18 years) active pulmonary TB patients (PTB) (n = 8), confirmed using solid or liquid TB culture, and non-TB controls (n = 8) provisionally diagnosed with PTB based on chest X-ray and other symptoms suggestive of PTB, but tested negative for smear microscopy and culture at enrolment and at two months follow-up. Serum samples were collected between July 2009 to December 2012 in Vietnam, before initiation of treatment. Information on TB genotype and status of latent or extrapulmonary TB (LTB, ETB) were unavailable. All human samples used in this study are from repositories for which subjects gave their informed consent for inclusion and are non-identifiable. The study was conducted with approval by the Alfred Ethics Committee (Certificate No. 169/13).

#### 2.3.2. Sodium Dodecyl Sulfate Polyacrylamide Gel Electrophoresis (SDS-PAGE) and Western Blot (WB)

Samples mixed with 2 × Laemmli sample buffer and 1 M dithiothreitol (95 °C, 10 min), and electrophoresed in NuPAGE 4–12% Bis-Tris Pre-cast gels (BioRad; Hercules, CA, USA) for 50 min at 150 V with Precision Plus Protein^TM^ Dual Color Standard (BioRad; Hercules, CA, USA) were dry-blotted using nitrocellulose membranes iBlot^®^ Gel Transfer Stacks (Invitrogen, Life Technologies; Carlsbad, CA, USA). Membranes were blocked by rolling in 5% skim milk in PBS-0.05% Tween-20 (Amresco; Solon, OH, USA) (RT, 1 h), followed by incubation in primary antibody from 1) pooled or individual (using a Mini-Protean Multiscreen apparatus (Biorad)) TB and non-TB sera at 1:1000; or 2) rabbit polyclonal anti-whole cell lysate (WCL) antibodies (NR-13819) at 1:5000 or pooled mouse monoclonal anti-LpqH (NR-13822), GroEL2 (NR-13790), PstS1 (NR-13790) antibodies from BEI Resources, NIAID, NIH at 1:500 (4 °C, overnight). This is followed by membrane incubation in HRP-labelled secondary antibodies (goat anti-mouse Ig (1:1000), swine anti-rabbit IgG (1:10,000), and rabbit anti-human IgG (1:5000)) (RT, 1 h). Finally, membranes were incubated in Luminata Forte Western HRP Substrate (Millipore; Bedford, MA, USA) (RT, 1 min) before imaging (CL-Xposure Film; Thermo Fisher Scientific). Membranes were washed thrice in PBS-0.05% Tween-20 between each incubation step.

### 2.4. Data Analysis

The MS/MS spectra data were used to identify proteins using the Mascot search algorithm (Matrix Science, London, UK) queried against Uniprot databases for MTB H37Rv (83332) and *M. bovis* BCG (410289), with trypsin set as the specific digest reagent. Significant protein matches (>2 significant peptide sequences) were annotated based on Gene Ontology (GO) terms, and analyzed using STRING 11.0 [35], a powerful web resource of known and predicted protein–protein interactions. As the BCG database was not available in STRING, we used the MTB H37Rv database for predictive protein-interaction analysis and to classify the identified proteins into their biological process and cellular compartment based on enriched GO terms. The Venn diagram online tool [36] was used to compare protein significant matches of A60-BCG S1 and S2, and to compare proteins of A60-BCG and EVs of MTB published by Lee et al. [37]. Data were tabulated and visualized using Microsoft Excel.

## 3. Results

### 3.1. Protein Identification and Annotation

**Figure 1:**
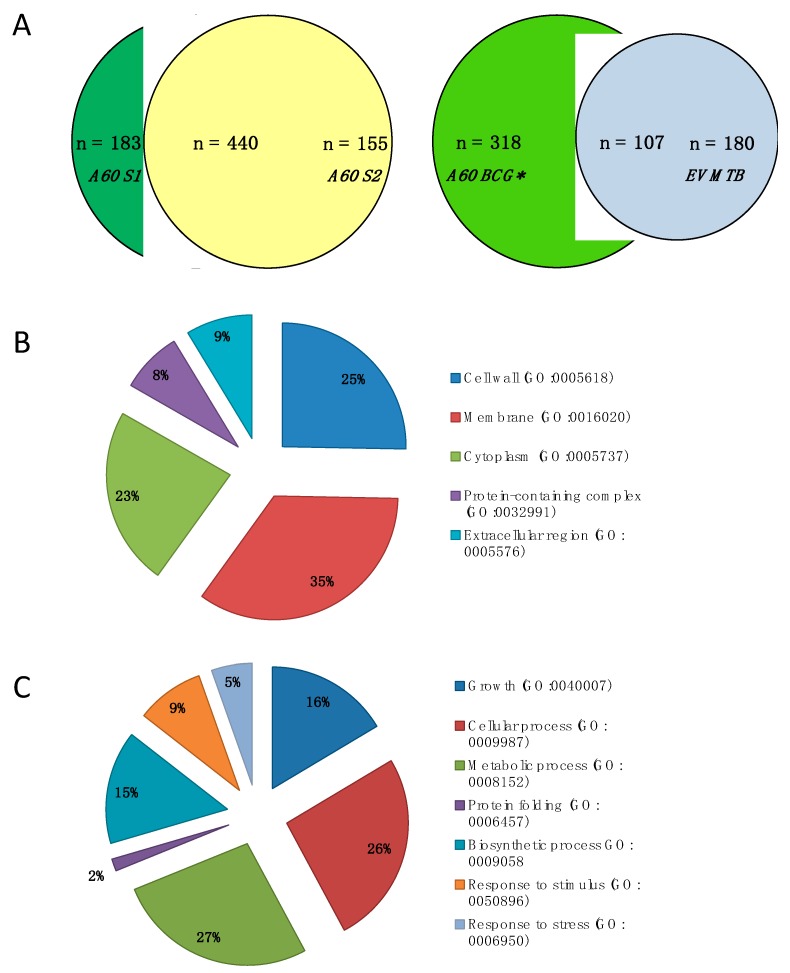
(**A**) Venn diagram of proteins identified using two samples of A60 as well as comparison of A60 of *M.bovis* Bacillus Calmette-Guérin (A60 BCG) and extracellular vesicles of *M. tuberculosis* (EV MTB) proteins. Pie charts show protein distribution based on (**B**) cellular component and (**C**) biological process based on Gene Ontology (GO) term functional enrichment on STRING analysis. A60 BCG * refers to the 426 shared proteins of A60 S1 and S2, with matches in MTB H37Rv Uniprot database.

Commercial A60 (S1 and S2) were subjected to shotgun proteomic analysis on LC-MS/MS and searched against BCG Uniprot database. Similar to previous studies of “method-antigen complexes” such as MTB and *M. bovis* PPD [38,39], shotgun proteomic analysis of the A60-BCG complex antigen preparation detected a high number of proteins, specifically 630 and 595 proteins with ≥2 significant matches in A60 S1 and A60 S2, respectively, of which 440 were common to both samples (71% of A60 S1; 74% of A60 S2), amounting to 778 combined protein matches (Figure 1A) (Table S1). Overall, the proteins identified in the A60 represents approximately 19.9% of the 3891 proteins identified in the BCG proteome [40].

Further analysis were conducted on the 440 proteins shared between A60 S1 and S2. Of these 440 proteins identified using BCG Uniprot database, 426 had matches in MTB H37Rv database. These 426 proteins were compared with the proteomic profile of EV MTB (Figure 1A) and annotated for GO terms based on enrichments in STRING analysis. Among A60 proteins with significant functional enrichment (FDR < 7.39 × 10^−5^), were those associated with metabolic processes (27%), cellular processes (26%), growth (16%) and biosynthetic processes (15%). For cellular components classification, a majority of the proteins were associated with the membrane (35%), followed by cell wall (25%), cytoplasm (23%), protein-containing/macromolecular complexes (8%) and extracellular regions (9%).

There were 107 common proteins between A60 BCG and EV MTB (Figure 1A, Table S2) constituting 37% of the EV MTB proteins published by Lee et al. (2015) [37]. Notably, the proteins identified in the A60 and EV MTB include several lipoproteins such as LpqN, LprG, LprF, LppX, LpqH, LprA and chaperone proteins such as HtpG, HSP16.3, DnaK/HSP70, GroEL1, GroEL2, ClpB, GroES. Several of the proteins detected in A60 such as DnaK, Wag31, LprG, AcpP, FbpA, Tpx, GroES, BfrA, PepA, and Mpt64, have also been found in different preparations of MTB PPD used in the Mantoux skin test [39]. 

### 3.2. Predictive Protein–Protein Interaction

**Figure 2:**
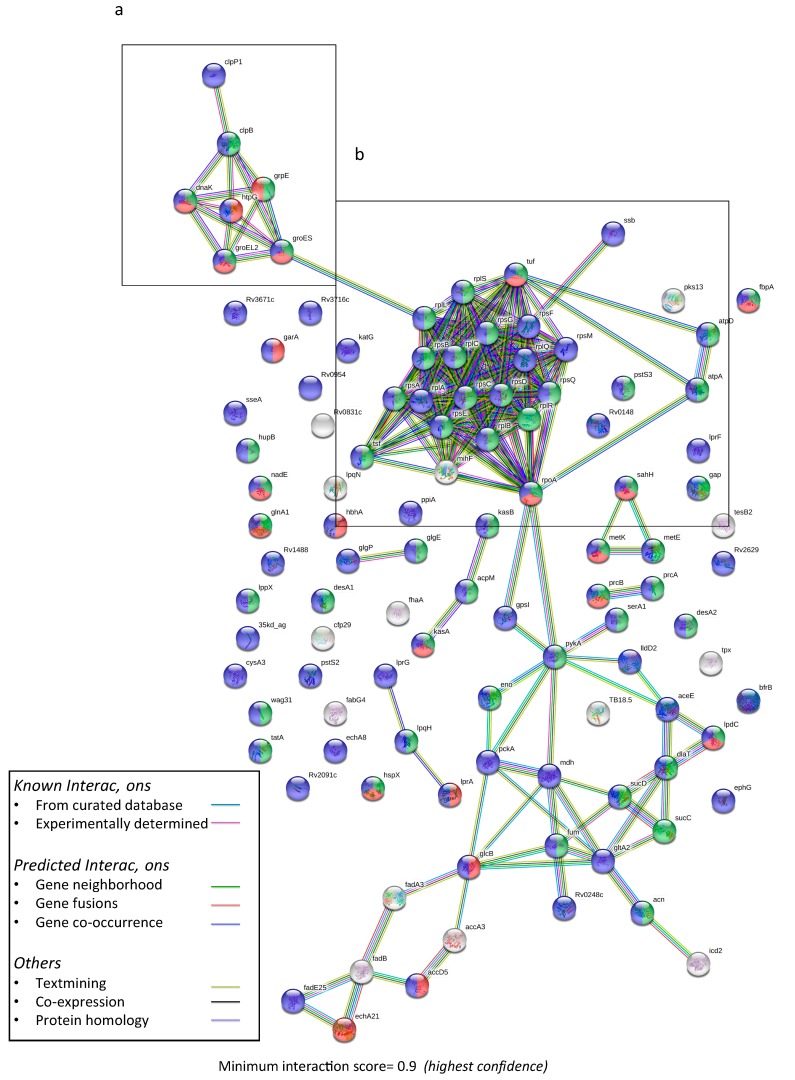
Predictive functional interaction network of 107 shared proteins of A60 and EV MTB using STRING 11.0, showing statistically significant functional interaction networks (*p*-value: <1.06 × 10^−16^) at minimum interaction score of 0.9. Boxes show (**a**) cluster of chaperone proteins and (**b**) cluster of ribosomal proteins. Node colors indicate functional enrichments based on GO:0040007 growth (green), GO:0005515 protein binding (red), and GO:0005886 plasma membrane (purple). EV MTB protein list was obtained from Lee et al., 2015 [37] (ProteomeXchange PXD001160).

Analysis of 426 proteins possessing MTB H37Rv homology using STRING MTB H37Rv database (set to highest confidence with interaction score of >0.9) showed significant predictive protein–protein interaction (PPI) enrichment (*p*-value: < 1.0 × 10^−16^), indicating that proteins in the A60 complex as a group are at least partially biologically connected. The main clusters of functional interactions observed were ribosomal/transcription and translation-related (29 proteins), metabolic enzymes (20 proteins), stress response/protein-refolding chaperones (12 proteins), and fatty acid biosynthesis (9 proteins) (Supplementary Figure S1). Significant interaction networks were also observed in the 107 proteins shared between A60 and EV MTB (*p*-value < 1.0 × 10^−16^), primarily consisting of plasma membrane proteins with functional enrichment for growth and protein binding, with clusters of chaperone proteins, ribosomal proteins, and enzymatic proteins (Figure 2).

Based on highest number of peptides with significant matches as well as experimental detection and available literature, 27 proteins of interest were also analyzed in greater detail (Table 1). The PPI enrichment analysis suggests significant functional interaction between the proteins (*p*-value 9.49 × 10^−11^) with three main clusters of interaction between (1) chaperonins DnaJ1, DnaK, GroEL1, GrpE, ClpB and GroEL2; (2) ligand transport-related LpqH and LprG; (3) ribosomal RpoB, RpoC, RpsA, Tuf, AtpA, and AtpD; and (4) fatty acid biosynthesis proteins Fas and FabG4. The highest scores obtained for predicted interaction were of rpoC with rpoB (0.999), atpD with atpA (0.999), GrpE with DnaK (0.998), and DnaJ1 with DnaK (0.998). Correspondingly, the top functional enrichments for these proteins were predicted to be protein folding (FDR: 1.17 × 10^−8^), growth (FDR: 7.59 × 10^−8^) and response to heat (FDR: 4.78 × 10^−7^), primarily related to chaperone protein activity and stress response. 

### 3.3. Antigenicity of A60-BCG

**Figure 3:**
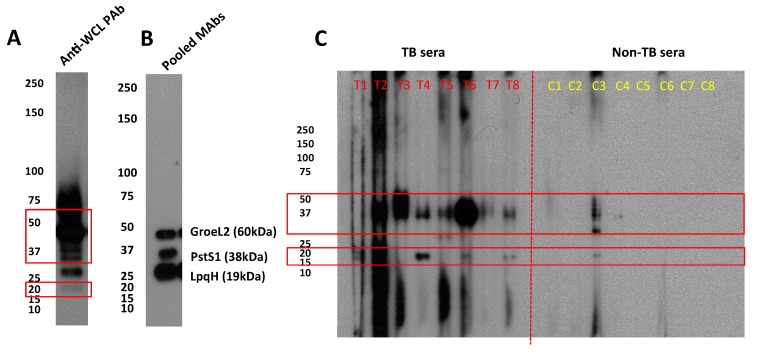
Proteins probed with (**A**) rabbit polyclonal anti-WCL antibodies, (**B**) pooled mouse monoclonal anti-GroEL2 (HSP 65), anti-PstS1 (38 kDa antigen), anti-LpqH (19 kDa antigen) antibodies, and (**C**) individual TB patient (T, n = 8) and non-TB control (C, n = 8) serum IgG.

Electrophoresed and blotted A60 probed with rabbit polyclonal anti-WCL antibodies showed several bands of varying intensity between 20 kDa and 75 kDa, with smeared bands approximately 50 kDa and higher, while clear bands were seen in lower molecular weight (MW) proteins between 20–37 kDa (Figure 3a). This pattern of antigenicity in rabbit antiserum appears similar when A60 was probed with patient sera samples. Western blot of individual serum IgG shows consistent reactivity against proteins approximately 37–50 kDa in MW were observed in 7/8 patients and 1/8 non-TB controls from Vietnam, while reactivity against a 20 kDa band was observed in 4/7 patients, and the same non-TB control (Figure 3c). Finally, strong bands were observed when probed using pooled mouse monoclonal anti-LpqH, anti-PstS1 and anti-GroEL2 antibodies, particularly for LpqH (Figure 3b). However, bands for PstS1 and GroEL2 appeared at lower MW while LpqH band appeared at a significantly higher MW.

## 4. Discussion

A60 is a large antigenic complex that has been consistently purified from lysate of log-phase grown BCG using a Sepharose 6B SEC column [34], albeit with some antigenic variation between batches. Despite its extended history, the origins of A60 and the reason this complex consistently appears upon cell lysis has not been investigated given that the micelle-forming complex was presumed to be an artefact of cell lysis. 

However, in recent years, there have been emerging interest in re-evaluation of classical mycobacterial antigen preparations using global approaches such as mass spectrometry, which may provide more insights into their characteristics to inform research in biomarkers and vaccine development. These studies have primarily focussed on the proteomic profiling of PPD [38,39], a cocktail of antigens used for determining latent tuberculosis in the Tuberculin Skin/Mantoux Test (including *M. bovis* PPD, used for diagnosis of bovine TB), which has been historically purified from steaming cultures of MTB by repeated precipitation with ammonium sulfate [41]. Given the crude nature of the PPD and A60, it is unsurprising that the two preparations reportedly share several similar antigens [25]. However, this is the first study known at present to proteomically characterize the A60 complex of *M. bovis* BCG including predictive protein–protein-interaction analysis of A60 members and their corresponding antigenicity.

Our MS results, identifying hundreds of different proteins, appear consistent with previous proteomic studies on complexes such as PPD reporting identification of 265 [39], 356 [38] and 608 [42] different proteins, and cellular component proteome studies such as *M. avium* and MTB cell wall identifying 309 [43] and 528 [44] different proteins. In this study, 778 unique proteins with ≥2 significant matches were identified in two experimental replicate A60 samples, among which 440 were detected in both samples while 183 and 155 proteins were unique to A60 S1 and A60 S2, respectively. Of the 440 proteins in A60, 426 proteins with MTB H37Rv homology were largely found to have significant enrichment for cell wall and membrane components, and functional enrichment for metabolic and cellular processes. This appears in line with the functional interaction networks observed on STRING analysis illustrating significant clustering among ribosomal proteins, metabolic enzymes, stress response/protein-refolding chaperones, and fatty acid biosynthesis. These same enrichments for proteins of growth and defense have also been reported in the extracellular proteins of other bacteria during the exponential/log-phase growth [45]. 

These findings suggest that despite assumptions that A60 complexes are artefacts of cell lysis and extraction methods, several proteins present in these heterogeneous complexes may possess physical or functional biological associations. This raises further questions whether the micellar structures that contain A60 proteins may be associated with proteins released extracellularly into EVs, since a total of 107 proteins identified in the A60 were also reported in Lee et al.’s (2015) proteomic analysis of EV MTB [37]. Several of these proteins such as LpqH, DnaK, GroEL1, and PstS1-related PstS3 [29,46,47], were found to be clustered with other chaperone proteins such as ClpB, DnaJ1 and GrpE based on predictive protein–protein-interaction analysis. The latter of which has been implicated as a novel immune activator capable of interacting with dendritic cells to generate Th1-biased memory T cells [48] and shown to confer better protection compared to that of DnaK-immunization [49]. Although the presence of several chaperones known to prevent misfolding, facilitate folding/refolding, and more recently, unfolding in order to recover functional proteins from aggregates appears in support of the A60 being a purposeful complex [50], the possibility of the protein aggregation being a product of bacterial cell lysis cannot be excluded [51]. Additionally, because DnaK and other chaperone proteins present in A60, such as HspX, and GroEL, are known to be highly homologous and conserved in different mycobacteria [52], it is unsurprising that attempts at using crude preparation such as A60 and PPD for diagnostic purposes have given rise to many false positive results [41].

Besides the presence of a chaperone protein cluster, other significant interactions found among 27 proteins of interest highlight clustering of protein synthesis-associated RpoB, RpoC, RpsA, Tuf, AtpA, and AtpD, and interaction between ligand transport proteins LpqH and LprG, which have been observed in EVs. While less is known about the latter three proteins, RpoB is well-described due to its association with resistance to the first-line TB drug rifampicin, with mutations in *rpoC* recently also found to influence RpoB-related resistance [53], while mutations for RpsA-coding genes is known to confer resistance against another first-line TB drug, pyrazinamide [54]. LpqH is a well-described protein consistently found in EVs, which has been observed to be overexpressed when EV production is increased [55], while LprG translocates lipoarabinomannan to the cell surface and transports triacylglycerides across the inner cell membrane into the periplasm [56]. Together these co-occurring ligand transport proteins are both virulence factors recognized by toll-like receptor 2 (TLR2) known to enhance TLR2-associated inflammation responses [47,57]. 

Despite the significant number of proteins identified on LC-MS/MS, experimental confirmation remains limited by existing monoclonal antibodies against the proteins. Using available antibodies, the Western blot analysis confirms presence of LpqH, GroEL2, and PstS1 likely due to molecular and functional similarity to PstS3 [31]. Although the protein bands appear at different molecular weights to their predicted sizes, particularly for LpqH, this size discrepancy has been observed previously and is hypothesized to relate to post-translational modifications and/or anomalous behaviour of the protein on SDS-PAGE [58]. 

When A60 was probed with TB patient sera samples, reactivity was most consistently observed against proteins approximately between 37–50 kDa and 20 kDa. These MWs appear to match the range of sizes of A60 proteins reported in earlier studies [15,21], as well as the sizes of the majority of the 27 proteins identified with the highest significant peptide matches on LC-MS/MS in this study. However, these were also the same bands recognized by a single non-TB control serum sample. This cross-reactivity may reflect the presence of conserved proteins such as DnaK and GroEL, and population exposure to environmental mycobacteria as is common in TB-endemic countries such as Vietnam, from which the serum samples were collected [59], although it is also possible that this patient may have had ETB that was not detected through the diagnostics methods utilised. 

The major limitations of the data presented relate to batch-to-batch variation of A60 and BCG, and the influence of sample processing for MS, which may hamper exact reproducibility of data. Although the analysis focussed on consistently detected proteins, as with many native antigenic preparations (including for EVs), contaminating proteins may be present which may result in artefacts. The STRING analysis is predictive and the interactions illustrated have yet to be experimentally validated, largely due to limitations in antibodies available against proteins detected, and lack of reactivity of available antibodies against certain proteins such as DnaK (data not shown) due to unknown reasons, which complicates attempts to verify predicted interactions through immunological methods such as Western blot and co-immunoprecipitation. Furthermore, due to limited databases available for *M. bovis* BCG, PPI analysis using STRING could only be conducted for proteins with peptide sequence homology to MTB H37Rv proteins. Finally, this analysis of A60 neglects the many glycolipid antigens that are not detectable by MS, and due to its qualitative nature, the proteins identified and their respective significant peptide matches provide limited information on protein abundance and distribution in the complex. Hence, in-depth quantitative studies are important to determine the abundance and presence of up- or down-regulated proteins in A60 especially for EV-associated proteins. Particularly striking is the signal intensity detected for LpqH on Western blot, a bonafide EV-associated protein, despite being detected with lower numbers of significant peptide matches—underscoring the qualitative nature of the MS analysis.

This study remains a first attempt to identify and characterize proteins present in A60 complex using a global proteomic bioinformatics approach, which has identified several proteins associated with MTB growth, survival and interaction with host immunity. Future work may focus on purification of A60-like HMW complexes from virulent MTB for comparison with HMW of attenuated/non-pathogenic strains, such as MTB H37Ra and BCG to identify upregulated proteins unique to MTB using labelled proteomic approaches such as Isobaric tags for relative and absolute quantitation (iTRAQ), and for direct proteomic and electron microscopic comparison with EVs. Such characterization may bring us closer to isolation of more consistent preparations of immunogenic and antigenic proteins which are specific to pathogenic MTB and possess reduced cross-reactivity with non-pathogenic mycobacteria, to inform the burgeoning research in mycobacterial EVs and impel progress in TB biomarker and vaccine development. 

## Figures and Tables

**Table 1:** Molecular and functional characteristics of proteins proteomically identified in A60.

No.	Protein Names	Gene Names	Length	Mass (Da)	Biological Process	Cellular Component	Peptide/Proteinin	
							Sig. Matches	Cover (%)
1	Chaperone protein DnaK	dnaK BCG_0389	625	66790	cellular response to stress, protein folding	bacterial extracellular vesicle	58	43.75
2	Probable fatty acid synthase (Fas)	fas BCG_2545c	3069	326790	metabolic process	cell wall, cytoplasm, plasma membrane	55	17.25
3	Chaperone protein ClpB	clpB BCG_0422c	848	92512	cellular response to stress, protein folding; metabolic process	cell wall, cytoplasm, plasma membrane	50.5	36.65
4	DNA-directed RNA polymerase subunit beta (RpoC)	rpoC BCG_0717	1316	147303	transcription	cell wall, cytoplasm, plasma membrane	46.5	33.35
5	Polyketide synthase (Pks13)	pks13 BCG_3862c	1733	186629	biosynthetic process	NA	46	21.7
6	Probable multifunctional mycocerosic acid synthase (Mas)	mas BCG_2962c	2111	225568	NA	NA	45.5	49.1
7	Cell wall synthesis protein Wag31	wag31 BCG_2162c	260	28260	cell wall synthesis	cell wall, plasma membrane	41	54.05
8	Probable succinate dehydrogenase	Rv0248c	646	71092	anaerobic respiration	cell wall, plasma membrane	39	34.9
9	35 kDa protein	35kd_ag BCG_2760c	270	29240	NA	cell wall, cytoplasm, plasma membrane	37.5	75.95
10	ATP synthase subunit alpha (AtpA)	atpA BCG_1368	549	59480	ATP production	cell wall, plasma membrane	37.5	23.7
11	Alpha-crystallin (HspX)	hspX BCG_2050c	144	16217	cellular response to stress, protein folding	cell wall, cytoplasm, plasma membrane	37	67.7
12	30S ribosomal protein S1 (RpsA)	rpsA BCG_1668	481	53199	translation	ribosome, cell wall, plasma membrane	36	38.65
13	ATP synthase subunit beta (AtpD)	atpD BCG_1370	486	53175	ATP production	cell wall, cytoplasm, plasma membrane	34	41.65
14	Elongation factor Tu (Tuf)	tuf BCG_0734	396	43566	response to stress, protein synthesis	cell wall, cytoplasm, plasma membrane	33	40.75
15	60 kDa chaperonin 2 (GroEL2)	groL2 groEL2 BCG_0479	540	56692	cellular response to stress, protein folding	cell wall, cytoplasm, plasma membrane	31	34.55
16	DNA topoisomerase 1 (TopA)	topA BCG_3704c	934	102307	DNA replication	cell wall, cytoplasm, plasma membrane	27.5	30.45
17	29 kDa antigen (CFP29)	cfp29 BCG_0850c	265	28870	defense response	NA	26	41.1
18	60 kDa chaperonin 1 (GroEL1)	groL1 groEL1 BCG_3487c	539	55843	cellular response to stress, protein folding	cell wall, cytoplasm, plasma membrane, nucleoid	23.5	32.5
19	Protein GrpE	grpE BCG_0390	235	24544	cellular response to starvation protein folding	cell wall, cytoplasm	22	28.95
20	DNA-directed RNA polymerase subunit beta (RpoB)	rpoB BCG_0716	1178	130354	transcription; response to antibiotic	cell wall, cytoplasm, plasma membrane	21.5	34.95
21	Chaperone protein DnaJ1	dnaJ1 BCG_0391	395	41746	cellular response to stress, protein folding	cell wall, cytoplasm, plasma membrane	21	23.95
22	Probable 3-oxoacyl-[acyl-carrier protein] reductase (FabG4)	fabG4 BCG_0280c	454	46916	NA	NA	20.5	47.05
23	Heparin-binding hemagglutinin (HbHA)	hbhA BCG_0516	199	21522	pathogenesis	cell wall, cytoplasm, plasma membrane	16	37.45
24	Phosphate-binding protein PstS3	pstS3 BCG_0980	370	38215	ligand transport; pathogenesis	Plasma membrane, secreted	10	21.75
25	Lipoarabinomannan carrier protein (LprG)	lprG BCG_1472c	236	24647	ligand transport; pathogenesis	bacterial extracellular vesicle	8.5	21.6
26	Glyceraldehyde-3-phosphate dehydrogenase (Gap)	gap BCG_1497	339	36105	metabolic process	cell wall, cytoplasm, plasma membrane	5	14.8
27	Lipoprotein LpqH	lpqH BCG_3822	159	15309	ligand transport; pathogenesis	bacterial extracellular vesicle	5	14.2

Note: Proteins 1–21 were selected and listed in order of decreasing number of significant peptide matches (average for A60 S1 and S2), while proteins 22–27 were selected based on importance according to existing literature. Asterisks indicate proteins that have been commonly found in proteomic analysis of all PPDs analyzed in Prasad et. al., 2013 [39].

## Supplementary Materials

The following are available online at https://www.mdpi.com/2076-393X/7/3/80/s1, (1) Table of total proteins identified, additional details (Table S1A,B) and 107 shared proteins of A60 and EV MTB (Table S2) (Excel). (2) Figure of protein networks among 426 proteins analyzed in STRING
