# Supplementary figures and images for: Proteomic Analysis of Antigen 60 Complex of M. bovis Bacillus Calmette-Guérin Reveals Presence of Extracellular Vesicle Proteins and Predicted Functional Interactions
Source: Vaccines (Basel). 2019 Aug 2;7(3):80. doi: 10.3390/vaccines7030080 (PMC6789874; doi:10.3390/vaccines7030080)

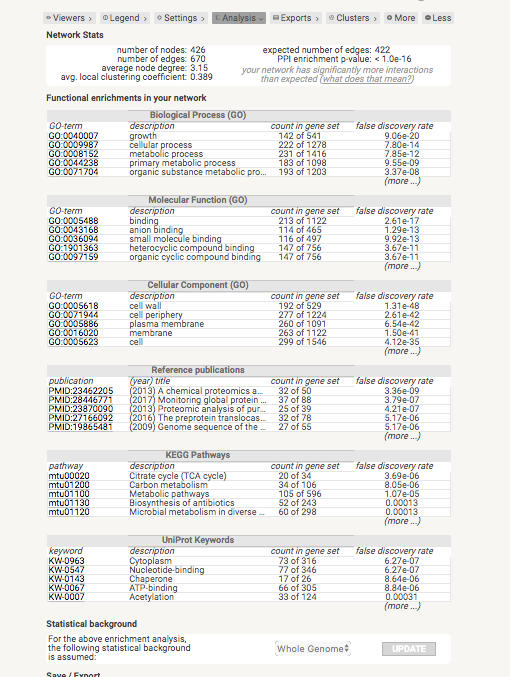

Supplement: Supplementary file 1 [file vaccines-07-00080-s001.zip › Figure S1b.png]
